# ADNP, a Microtubule Interacting Protein, Provides Neuroprotection Through End Binding Proteins and Tau: An Amplifier Effect

**DOI:** 10.3389/fnmol.2018.00151

**Published:** 2018-05-01

**Authors:** Illana Gozes, Yanina Ivashko-Pachima, Carmen L. Sayas

**Affiliations:** ^1^The Lily and Avraham Gildor Chair for the Investigation of Growth Factors, Dr. Diana and Zelman Elton (Elbaum) Laboratory for Molecular Neuroendocrinology, Department of Human Molecular Genetics and Biochemistry, Sackler Faculty of Medicine, Sagol School of Neuroscience and Adams Super Center for Brain Studies, Tel Aviv University, Tel Aviv, Israel; ^2^Centre for Biomedical Research of the Canary Islands, Institute for Biomedical Technologies, Universidad de La Laguna, Tenerife, Spain

**Keywords:** microtubules, microtubule-associated proteins, ADNP, tau, microtubule end binding proteins

## Activity-dependent neuroprotective protein (ADNP) interacts with microtubules

Neuronal plasticity, key to brain function in health, is impaired in neurodevelopmental, neuropsychiatric and neurodegenerative diseases. Neuronal plasticity depends on an intact cytoskeletal system. Here, we focus on recently discovered as well as “classical” cytoskeletal proteins including activity-dependent neuroprotective protein (ADNP), Tau and microtubule end binding proteins (EBs), impacting neuroplasticity and neuropathology.

Original structure-function analysis of astrocyte-secreted protein fragments identified femtomolar-acting neuroprotective peptide moieties VLGGGSALLRSIPA (Brenneman and Gozes, 1996), SALLRSIPA (Brenneman et al., 1998) and NAPVSIPQ (Bassan et al., 1999), with NAPVSIPQ (NAP) being a fragment of ADNP (Bassan et al., 1999). However, ADNP does not only provide neuroprotection through the NAP motif, but is essential for brain formation, with complete *Adnp* gene knockout in mice resulting in neural tube closure failure and fetal death. Furthermore, NAP promotes neural tube closure in the face of alcohol intoxication (Chen et al., 2005). Thus, the mechanism of ADNP protection, potentially through the potent NAP motif, is of interest.

In search for NAP binding partners, we subjected mouse brain protein extracts to affinity chromatography with NAP as a ligand and identified tubulin as an interacting partner (Divinski et al., 2004, 2006). These results were coupled to NAP promoting changes in microtubule structure and protecting against microtubule disassembly induced by nocodazole *in vitro* (Gozes and Divinski, 2007) and colchicine *in vivo* (Jouroukhin et al., 2013). Further data suggested NAP protection against Zinc intoxication, which was originally linked to microtubule disruption (Divinski et al., 2004, 2006) and increased specificity to beta III tubulin, or to neuronal cells (Divinski et al., 2006; Holtser-Cochav et al., 2006). Parallel studies identified reduced axonal transport (Amram et al., 2016), increased tau hyperphosphorylation and tau depositions as a consequence of *Adnp* haploinsufficiency (Vulih-Shultzman et al., 2007). However, direct interaction of NAP with pure tubulin was not confirmed (Yenjerla et al., 2010). Thus, the discovery of (1) the requirement for the SIP motif on NAPVSIPQ and related peptides for neuroprotection (Wilkemeyer et al., 2003), (2) the SxIP microtubule end binding protein 1 (EB1) interacting motif as a microtubule tip localization signal (Honnappa et al., 2009; Jiang et al., 2012), and (3) EB3 as essential for dendritic spine formation(Jaworski et al., 2009), directed research toward ADNP-NAP-EB1/3 interactions. EB1/3 proteins are the master regulators of the microtubule plus-end tracking proteins (+TIPs), which accumulate at the growing ends of microtubules, showing a “comet” pattern at microtubule tips (Lansbergen and Akhmanova, 2006).

## The identification of the NAP/ADNP EB-direct interaction and EB requirement for NAP activity

To establish a direct connection between ADNP and the EB family of proteins, specific immunoprecipitation experiments were carried out, showing direct interactions and enhancement of EB3-ADNP as well as other microtubule plus-end protein interactions by NAP (Oz et al., 2014). Further affinity chromatography with NAPVSIPQ and recombinant EB proteins (Oz et al., 2014) showed direct interaction with NAP and identified displacement with NAPVSKIPQ (SxIP = SKIP), but not with NAPVAAAAQ. These studies were further elaborated to show direct interactions in COS-7 cells expressing fluorescent EB3 and subject to fluorescent-NAP. Finally, silencing of EB1 and EB3, but not of EB2, abolished NAP protection in the neuronal model pheochromocytoma (PC12) against Zinc intoxication (Oz et al., 2014), and NAP increased PSD-95 expression in dendritic spines, which was inhibited by EB3 silencing (Oz et al., 2014). Together, these studies implicate ADNP/NAP in synaptic plasticity, involving EB proteins (Jaworski et al., 2009; Oz et al., 2014). While these results explained the previously observed NAP interaction with microtubules, bringing into focus the SIP motif (Gozes et al., 2016; Quraishe et al., 2016) and suggesting an amplifier effect at the microtubule tip, the molecular mechanism of increased Tau hyperphosphorylation, as a consequence of ADNP deficiency and protection by NAP against tauopathy (Vulih-Shultzman et al., 2007; Matsuoka et al., 2008; Shiryaev et al., 2009; Jouroukhin et al., 2013), still required further investigations.

## Tau regulates the localization and function of EB1 and EB3 in developing neuronal cells and antagonizes EB tracking at microtubule ends through a phosphorylation-dependent mechanism

Tau and EBs were shown to partially co-localize at extending neurites of N1E-115 neuroblastoma cells and axons of primary hippocampal neurons, confirmed by immunoprecipitation and by tau/EB1 direct *in vitro* pull-down assays (Sayas et al., 2015). Fluorescence recovery after photobleaching assays performed in neuroblastoma cells corroborated tau modulation of EB3 cellular mobility (Sayas et al., 2015). Another excellent report shows that Tau and EBs form a complex via the C-terminal region of EBs and the microtubule-binding sites of Tau and further show that these two domains are required for the inhibitory activity of Tau on EB localization to microtubule ends. Additionally, their results show that the phosphomimetic mutation S262E within Tau microtubule-binding sites impairs EB/Tau interaction and prevents the inhibitory effect of Tau on EB comets (Ramirez-Rios et al., 2016). The question then arose if there is an EBs/Tau-ADNP/NAP connection.

## ADNP/NAP dramatically increase microtubule end-binding protein-tau interaction: a novel avenue for protection against tauopathy

**Figure 1:**
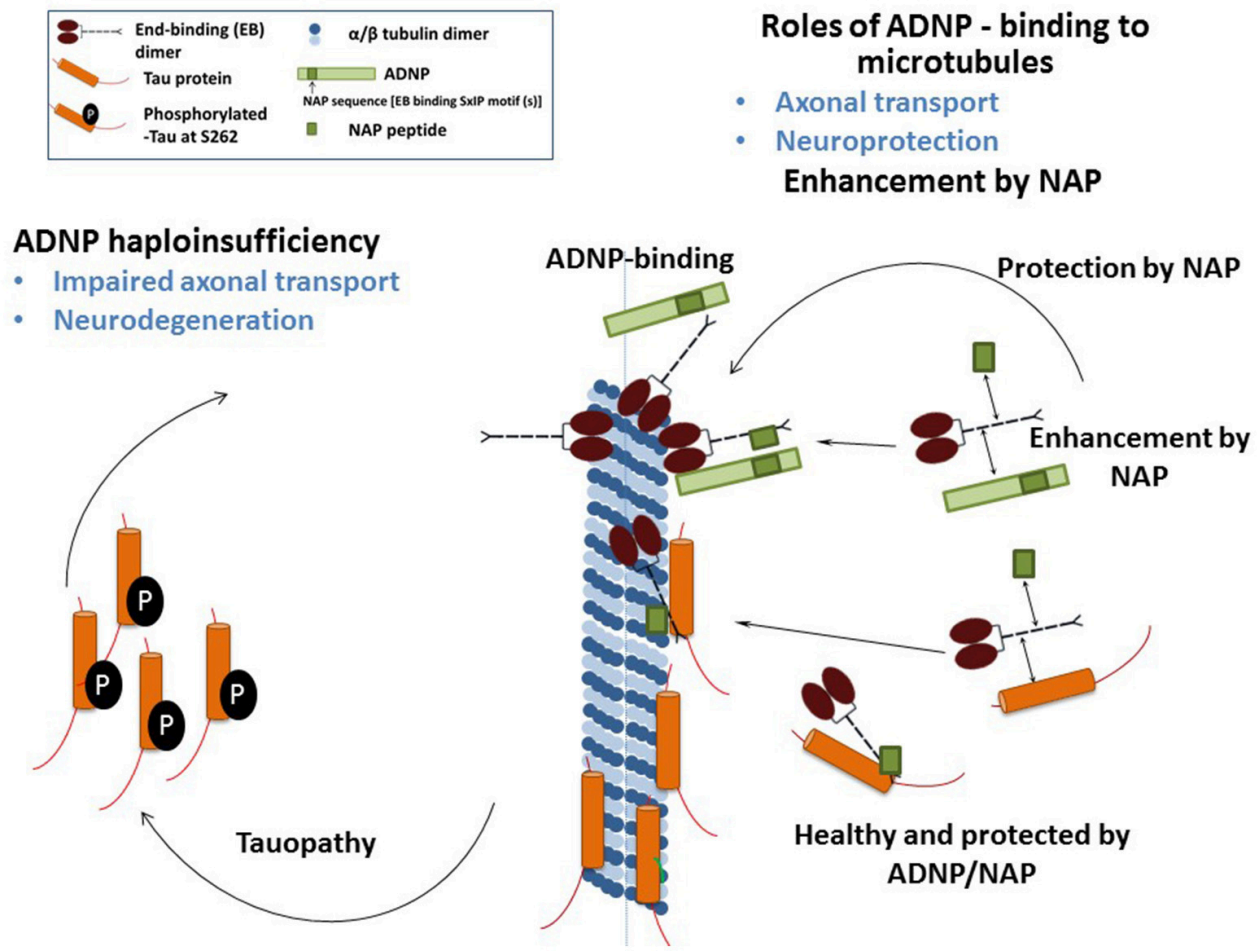
The illustration shows the mechanism of NAP/ADNP neuroprotection against tauopathy in the disease state. In the healthy state, ADNP enhances axonal transport and synaptic plasticity. ADNP has two adjacent SIP motifs, one within the NAP sequence and one upstream (Bassan et al., 1999; Zamostiano et al., 2001). A NAP—related peptide SKIP (encompassing the SxIP motif), enhances axonal transport in *Adnp* haploinsufficient mice, probably through the same mechanism (Amram et al., 2016). In tissue culture cells, NAP also enhances dendritic spine formation (synaptic plasticity) through an EB3-depedent mechanism (Oz et al., 2014).

We have recently demonstrated that NAP augmented EB1 and EB3 comet density, amounts, length and speed in the N1E-115 neuroblastoma neuronal model. NAP enhanced EB3 homodimer formation, while decreasing EB1-EB3 heterodimer content and driving EB1- and EB3-Tau interactions (dramatic 20-fold increases), leading to recruitment of EB1/EB3 and Tau to microtubules under zinc intoxication, which has previously been shown to be linked to Tau hyperphosphorylation (Ivashko-Pachima et al., 2017). As indicated above, our previous results showed that while NAP protected neuronal-like cells against oxidative stress, it did not protect NIH3T3 fibroblasts (Divinski et al., 2004). Indeed, NAP did not protect NIH3T3 cells against zinc intoxication, unless these cells were transfected with Tau. Interestingly, other microtubule-associated proteins (MAPs) may replace Tau; thus, EB-Tau (MAPs) interaction is identified as a novel target for endogenous ADNP neuroprotection (Ivashko-Pachima et al., 2017). Importantly, as indicated, phosphorylation of S262 impaired EB/Tau interactions and our previous data have directly shown that NAP inhibits tau hyperphosphorylation at the S262 site (Jouroukhin et al., 2013) in multiple tauopathy models (Magen et al., 2014), with this phosphorylation site being linked to impaired axonal transport and neurodegeneration (Iijima-Ando et al., 2012), partially solving the NAP/ADNP protective activity against tauopathy (Figure 1). Future studies encompassing the impact of the NAP-EBs-Tau interaction on Tau aggregation (Gozes et al., 2014a,b), mitochondrial function (Esteves et al., 2014) and autophagy (Sragovich et al., 2017) are planned and will contribute to clarify the relevance of this protein complex in neuroprotection in the context of tauopathies and other neurodegenerative diseases.

## The ADNP syndrome

The ADNP syndrome (Helsmoortel et al., 2014), a recently described autism spectrum disorder syndrome driven by heterozygous, mostly protein truncating, *de novo* mutations in ADNP (Gozes et al., 2015, 2017a,b), is a subject of our future studies. These studies are aimed at connecting protein structure to function, with the human condition being characterized with intellectual disability, global developmental delays (including motor delays) and facial dimorphisms. Interestingly, ~80% of the ADNP children can be identified by premature deciduous tooth eruption, a unique early diagnostic marker. Teething and bone/brain formation converge on mechanisms linked to ubiquitin impacted by the cytoskeleton, paving the path to future research. From a clinical perspective, Coronis Neurosciences (www.coronisns.com) is developing NAP (CP201) (Magen and Gozes, 2013, 2014) for the ADNP syndrome.

## Author contributions

All authors listed, have made substantial, direct and intellectual contribution to the work, and approved it for publication.

### Conflict of interest statement

The authors declare that the research was conducted in the absence of any commercial or financial relationships that could be construed as a potential conflict of interest.
